# Gastrointestinal Conditions Affect Chronic Pain and Quality of Life in Women

**DOI:** 10.3390/ijerph21111435

**Published:** 2024-10-29

**Authors:** Ilenia Casini, Lauretta Massai, Erminia Solomita, Kathleen Ortenzi, Stefano Pieretti, Anna Maria Aloisi

**Affiliations:** 1 Pain and Stress Neurophysiology Laboratory, Department of Medicine, Surgery and Neuroscience, University of Siena, Via Aldo Moro 2, 53100 Siena, Italy; ilenia.casini@student.unisi.it (I.C.); lauretta.massai@unisi.it (L.M.); erminia.solomita@unisi.it (E.S.); kathleen.ortenzi@unisi.it (K.O.); 2 National Centre for Drug Research and Evaluation, Istituto Superiore di Sanità, Viale Regina Elena 299, 00161 Rome, Italy; stefano.pieretti@iss.it

**Keywords:** chronic pain, gastrointestinal disorders, dietary inflammatory index

## Abstract

Pain is a chronic condition in many women; drugs used for its treatment are often accompanied by detrimental effects on many organs, including the gut. Once inflamed, the gut can affect pain processes. The aim of this study was to evaluate the general health of women suffering chronic pain, with particular attention to gastrointestinal (GI) conditions. The possibility to improve pain and quality of life through personalized nutritional advice was also tested. Forty women suffering from chronic pain were contacted for the administration of questionnaires to define their pain features and gastrointestinal conditions. Their psychological, clinical and reproductive states were also recorded. Pain scores were correlated with GI, psychological and clinical scores. Diet suggestions were given, and evaluation was repeated after 4 weeks. Thirty-eight women were included in the study: 32 suffered chronic widespread pain and had 6 pelvic pain. Pain had been present in all women for years; more than 80% of women reported various types of disorders related to the gut. Pain scores were worse in the women intolerant to milk and dairy products. The GI score was positively correlated with the pain score. The Dietary Inflammatory Index was very high in all subjects. Personalized nutritional advice followed by 26 subjects for 4 weeks resulted in a significant improvement of pain and quality of life parameters. We describe women with chronic pain as being particularly affected by GI alterations. The change in feeding habits had a beneficial effect on pain and other quality of life parameters.

## 1. Introduction

We recently reported that chronic pain can be significantly improved with changes in feeding habits. IgG4 dosage was used to determine specific food intolerances, and the exclusion of these foods was able to decrease the pain intensity [1]. Moreover, a personalized Mediterranean diet (known to have an anti-inflammatory action) was shown to improve the pain and quality of life in fibromyalgia patients [2]; it became evident that their pain was directly related to certain foods and thus to their interaction with the gut. In chronic pain patients, it is highly possible that once the chronic pain has had a long duration, and many drugs have been used, the gut functions can be significantly affected, resulting in gastritis, constipation, diarrhea, bloating and inflammation with structural alterations [3]. It also must be considered that some primary gut diseases are often present in chronic pain patients. For instance, it has repeatedly been reported that a high percentage of fibromyalgia patients complain of irritable bowel syndrome (IBS) [4,5].

The gut does not have a pain system able to send detailed information about inflammation and/or lesions; thus, for patients with GI disorders, there are only mild signs, not always able to indicate the magnitude of the problem and its localization. Signs of discomfort like abdominal swelling, diarrhea, constipation, gastroesophageal reflux, etc., are common and are generally treated with symptomatic drugs. In many subjects, these signs can be considered ‘normal’ due to familial or social habits. Only recently has it become clear that these conditions could be accompanied by serious disruption of intestinal functions: for example, the loss of its impermeability [6] could increase the interactions of bacteria with the host’s immune system and the release of inflammatory markers like cytokines [7].

The aim of the present study on women with chronic pain was to evaluate if their painful condition was accompanied by altered gastrointestinal, nutritional, psychological and dietary factors and to determine if directing their diet towards anti-inflammatory foods would contribute to improving the recorded parameters. In the present study, the nutritional advice given to each subject was based on the Mediterranean diet [8,9], but fully personalized for each patient due to allergies, intolerances, eating habits, etc.

## 2. Subjects and Methods

Women suffering nononcological chronic pain were recruited by means of online social media and local advertising. Subjects were contacted to schedule the visit (Visit 1) in person or by teleconferencing. During the visit, each patient was asked to fill out questionnaires, to submit anthropometric measurements (weight and height) and, if in person, to carry out bioelectrical impedance analysis (BIA).

Nutritional habits were evaluated for each patient by way of questions about the daily habitual mealtime and a food diary of the previous day in order to calculate the intake of energy, macro- and micronutrients.

The experimental protocol adhered to the principles of the Declaration of Helsinki of 1964 and its later amendments; the experimental procedure was approved by the University of Siena Local Ethics Committee (CAREUS, 7/2020 of 15/09/2020). Once included in the study, each subject provided informed consent for the analysis and publication of the data.

**Figure 1:**
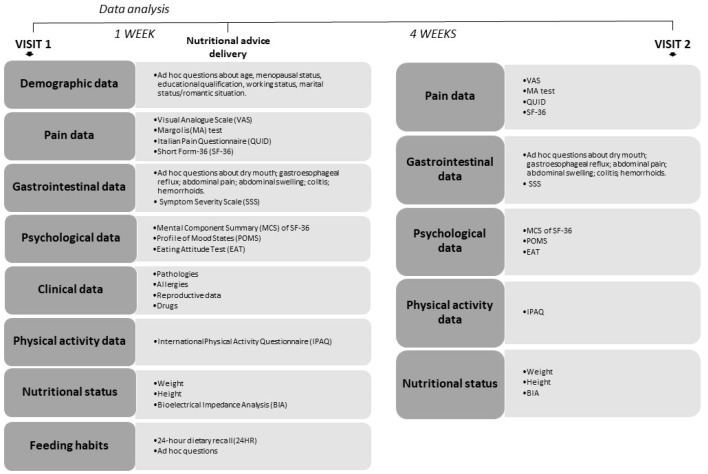
Experimental procedure, list of tests/questionnaires administered during Visit 1. In the subjects that followed the nutritional suggestions, the same procedure was repeated after 4 weeks (Visit 2).

The experimental design is shown schematically in Figure 1.

## 3. Experimental Procedure

(1) Visit 1 and Data Analysis: a qualified researcher met the subjects, in person or by teleconferencing, to collect the anamnesis, including pain/pains, familial, social, environmental, work and gastrointestinal conditions; if possible, anthropometric measurements and BIA were carried out. Feeding data were analyzed to evaluate the kind/amount/frequency/consistency of meals. A personalized list of nutritional advice was given to the subject, who was invited to meet again after 4 weeks.

(2) Visit 2: the determinations were repeated.

Each subject was asked to fill out the following questionnaires regarding:

**Demographic data** (age, menopausal status, educational qualification, working status, marital status/romantic situation).

**Pain.** Four questionnaires were used to build pain scores able to focus on different pain qualities:

The Visual Analogue Scale (VAS) is a unidimensional measure of pain [10,11]. The mean of the three determinations (m3) was used and reported as **m3VAS**. As described in Table S1, m3VAS was divided into four ranges, receiving a score from 0 (best condition) to 3 (worst condition).

Margolis (MA) is a drawing pain rating system that evaluates 45 anatomical areas each with a corresponding percentage value (0–100) of body surface in which is present pain in order to compute a total weighted score of body surface in pain [12]. The mean of the three determinations was used and reported as **m3MA**. As described in Table S1, the m3MA data were divided into four ranges, receiving a score from 0 (best condition) to 3 (worst condition).

The Italian Pain Questionnaire (QUID) was used to study the qualitative aspects of pain experienced in the last week [13]. It consists of 42 pain descriptors divided into four main classes: sensory (S), affective (A), emotional (E) and miscellaneous (M), their sum being used to build the Pain Rating Index rank-Total (PRIr-T). PRIr-T was divided into four ranges, receiving a score from 0 (best condition) to 3 (worst condition), as reported in Table S1.

The Bodily Pain (BP) scale present in the SF-36 questionnaire was used to evaluate the effect of pain on general health [14]. As described in Table S1, the BP values were divided into four ranges, receiving a score from 0 (best condition, higher values) to 3 (worst condition, lower values).

Thus, the overall pain score ranges from 0 (best condition) to 12 (worst condition).

**Gastrointestinal (GI) condition.** As reported in Table S1, the GI score was based on the presence of the following conditions: dry mouth, gastroesophageal reflux, abdominal pain, abdominal swelling, colitis and hemorrhoids. Each parameter was scored as the presence/no presence (YES: score 0, NO: score 1) of the condition. Thus, the GI score can vary from 0 (best condition) to 6 (worst condition).

**GI Symptom Severity Scale (SSS)** was specifically designed to assess the severity of IBS in a patient [15]. In the SSS total score, mild, moderate and severe cases were indicated by values of 90 to 210, 210 to 360 and > 360, respectively. For the analysis of this questionnaire, we used the total scale and subscales individually (API, APF, AS, AH, ES, IQL).

**Psychological condition.** A psychological score was built as a sum of values from:

Mental Component Summary (MCS) of the SF-36 questionnaire. It includes four scales, vitality (V), social functioning (SF), emotional role (ER), and mental health (MH). Each scale is assigned a score from 0 to 100, where a higher score means better health. As described in Table S1, the MCS values were divided into four ranges, receiving a score from 0 (higher values, best condition) to 3 (lower values, worst condition).

Profile of Mood States (POMS), widely used to assess transient, distinct mood states and mood changes [16]. As reported in Table S1, for each scale, if the value was in the healthy range a score of 0 was assigned, while a score of 1 was given for values outside the healthy range. Thus, the final POMS score can vary from 0 (best condition) to 6 (worst condition).

The Eating Attitude Test (EAT) is sensitive to the presence of an eating disorder [17]. EAT consists of 26 questions to which the subject can answer: always (3), usually (2), often (1), sometimes (0), rarely (0), never (0). If the sum is ≥ 20, an eating disorder can be suspected. As reported in Table S1, values lower than 20 were marked with 0 (best condition), values higher than 20 were marked with 1 (worst condition). Thus, the psychological score can vary from 0 (best condition) to 10 (worst condition).

**Clinical condition.** Illnesses and/or disorders acknowledged by the subjects were used to calculate a clinical score: in particular, endocrinopathies related to pancreas or thyroid, sleep features (sleep quality, insomnia, frequent awakenings, snoring), cardiovascular/lymphatic functions (lymphatic disorders, vasculopathy, cardiac alterations, anemia, blood pressure, plasma cholesterol) and genito-urinary functions (kidney disorders, bladder cystitis, vaginal candida). As reported in Table S1, each condition was scored as the presence/no presence (YES: score 0, NO: score 1) of the condition. Thus, the clinical score can vary between 0 (best condition) and 15 (worst condition).

**Reproductive condition.** The following conditions were considered to calculate a reproductive score: painful menstrual cycle, heavy menstrual cycle, irregular menstrual cycle, spontaneous abortions, pregnancies, use of contraceptives, presence of menopause symptoms, use of hormonal therapy, breast and ovarian-uterus pathologies. As reported in Table S1, each parameter was scored as the presence/no presence (YES: score 0, NO: score 1) of the condition. Thus, the reproductive score can have values between 0 (best condition) and 10 (worst condition).

**Allergy condition.** The following conditions were considered to build an allergy score: drug allergies, environmental allergies and food allergies. Each parameter was scored as the presence/no presence (YES: score 0, NO: score 1) of the condition and the score ranges from 0 (best condition) to 3 (worst condition) (Table S1).

**Drug use.** Consumption of the following classes of drugs was used to build a drug score: gastroprotective products, cortisones, opioids, cannabinoids, nonsteroidal anti-inflammatory drugs (NSAIDs), antipyretics, antidepressants, analgesics, muscle relaxants and sedatives. As reported in Table S1, consumption or nonconsumption were scored (YES: score 0, NO: score 1), and thus the drug score can vary from 0 (best condition) to 10 (worst condition).

**Physical activity.** The international physical activity questionnaire (IPAQ) was used to calculate a physical activity score [18]. As reported in Table S1, for each type of activity, a score of 0 was assigned to those who did an activity for at least 30 min per week, and a score of 1 to those who did not do any activity or who did an activity for less than 30 min. Thus, the physical activity score can vary from 0 (best condition) to 3 (worst condition).

**Body Mass Index (BMI)** was obtained from the measures of weight and height using the Quetelet equation [body mass (kg)/height^2^ (m^2^)] [19].

**Nutritional status.** This was determined only in women examined in person due to the possibility to carry out the BIA exam; the list of subjects met in person is present in Table 2. In those subjects, the BMI and BIA values were used to build a nutritional score:

BMI data were scored as follows: 0-normal weight (BMI 18–25), 1-overweight (BMI 25–30) and 2-obese (BMI 30+) (Table S1).

BIA was used to estimate body composition with a bioimpedentiometer (Akern Srl, Florence, Italy; [20]. Data were divided into two ranges depending on whether or not they fell within the reference values (YES: score 0, NO: score 1). Thus, the body composition score can vary from 0 (best condition) to 4 (worst condition) (Table S1).

The nutritional score ranges from 0 (best condition) to 6 (worst condition).

**Feeding habits** of the participants were investigated regarding the composition of meals taken during the day, daily water intake, presence of fruit and vegetables within the day, and weekly fish consumption. The 24-h dietary recall (24HR) was used to investigate baseline dietary intake [21]. It is based on the interview concerning all foods and beverages consumed by the respondent in the past 24 h. MetaDieta Software (METEDA, Version Professional 4.0.1, Rome, Italy) was used to analyze 24HR. The 24HR data were used to calculate an overall **Dietary Inflammatory Index (DII)**. In particular, 27 dietary parameters were extrapolated: alcohol, β-carotene, cholesterol, carbohydrates, energy, fats, fiber, folic acid, iron, magnesium, zinc, vitamin A, vitamin B-6, vitamin B-12, vitamin C, vitamin D, vitamin E, monounsaturated fatty acid, protein, niacin, riboflavin, (*n*-3) fatty acids, (*n*-6) fatty acids, polyunsaturated fatty acids, saturated fat, selenium and thiamine. The calculation of DII followed previously published protocols [21]. The individual overall DII was the sum of 27 food parameter-specific DIIs. The DII can vary from −5.5 (anti-inflammatory range, best condition) to +5.5 (proinflammatory range, worst condition).

The list of personalized nutritional advice was designed by a team of nutritionists based on each patient’s data, including BMI, age, physical activity and food-related allergies (if reported), as suggested by the Dietary Reference Values of Nutrients and Energy for the Italian population (LARN IV Revision) [22]. The daily intake of nutrients was based on the Mediterranean diet pattern, whose anti-inflammatory properties are known [23,24,25].

**Statistical analysis.** After a data normality check (Kolmogorov–Smirnov test, *n* > 30 and Shapiro–Wilk W test, *n* ≤ 30), the data were analyzed with analysis of variance (ANOVA) (normal data) or with the Kolmogorov–Smirnov test (non-normal data) carried out with several factors, including pain (two levels: chronic widespread pain, CWP, and pelvic pain, PP), menopause (two levels: premenopausal women, pre-MW, and menopausal women, MW), Test (two levels: Visit 1 and Visit 2) and Milk (two levels: diet with milk, DIET, and diet without milk, DIET NM). Details are given in the different parts of the Results section. Post-hoc analysis was carried out by Fisher’s least significant difference (LSD) test when necessary. Correlations were carried out with Pearson correlation coefficients (normal data) or with Spearman correlations (non-normal data). A *p* < 0.05 was considered significant. All analyses were performed with Statistica (StatSoft Inc., Tulsa, OK, USA) software. Data are reported as the mean and standard error of the mean (SEM).

## 4. Results

Forty women were considered and 38 were included in the study; all subjects gave their informed consent and completed all experimental procedures related to Visit 1. Demographic data are reported in Table 1. Data were collected from June 2020 to May 2022. Due to logistic/sanitary (COVID-19) problems, 11/40 of the subjects were contacted remotely.

The questionnaires were analyzed, and the data were scored as detailed in Table S1. ‘Scoring’ of the different parameters was chosen in light of the high number of data points and the need to group parameters belonging to the same symptom/class. Some parameters, like BMI, were used singly and/or grouped due to the need to make a particular analysis/correlation.

**Pain score.** Chronic pain (not related to the gut) had been present in all women (*n* = 38) for years (range 2–25). Based on the kind of pain/pains suffered, the subjects could be divided into two main groups: chronic widespread pain (CWP) group (*n* = 32) and pelvic pain (PP) group (*n* = 6). The pain values considered were those attributed to the main painful condition. Indeed, in many subjects more than one pain was present; in particular, 13 women reported back pain, 5 disc herniations, 14 pain in the lower limbs, 10 pain in the upper limbs and 7 in the neck. Moreover, 5 women reported osteoarthritis and 6 arthritis. In addition, some women reported neuralgia, tendon pain, recurring cramps, paresthesia and hypersensitivity.

As reported in Table S2, 33/38 women reported m3VAS values (mean of the three daily determinations) greater than 4; of the latter, 7/33 reported values equal to or higher than 8. For MA, used to evaluate the percentage of the body area affected by pain, the mean of the three daily determinations (m3MA) showed that 11/38 subjects had more than 25% of the body in pain; of the latter, 6/11 reported values equal to or higher than 40%. The PRIr-T represents the sum of the QUID subscales data. It indicates the quality of pain, and a higher score means a worse condition. In this population, 12 subjects had a PRIr-T value greater than 24 (average value for healthy people); of the latter, 6/12 had a value above 37 (average value for chronic pain syndromes).

The BP scale assesses pain frequency and pain interference with usual activities. The results showed that 12/38 subjects had values lower than 25 (worse condition).

**Figure 2:**
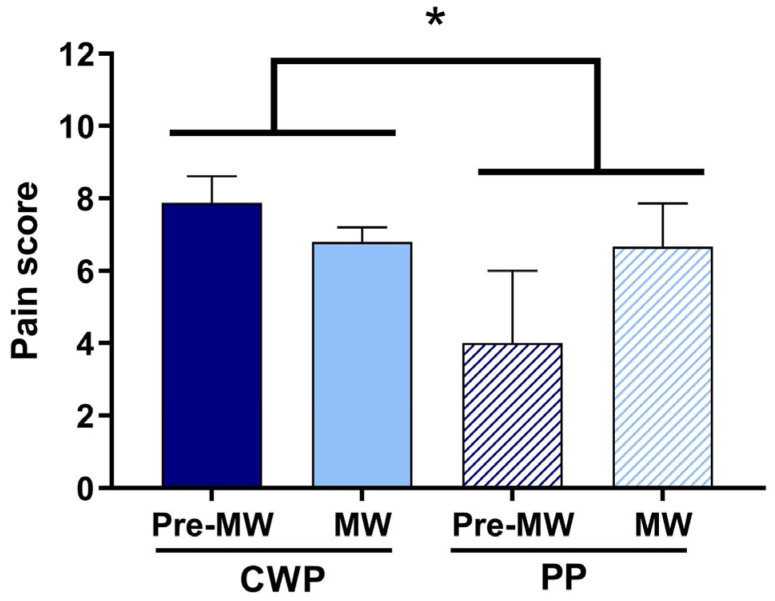
Pain score in the four groups. Women were divided into four groups depending on the menopausal condition (reproductive period, pre-MW, *n* = 11; menopausal period, MW, *n* = 27) and type of pain (chronic widespread pain, CWP, *n* = 32; pelvic pain, PP, *n* = 6). * *p* < 0.05 PP vs. CWP. Data are reported as mean ± SEM.

ANOVA applied to the pain score determined in all women with the factors menopause (pre-MW, MW) and pain (CWP, PP) revealed a significant effect of pain (F(1,34) = 4.09, *p* < 0.05) due to CWP having higher levels than PP independently of the menopause condition (Figure 2, Table 2).

**Figure 3:**
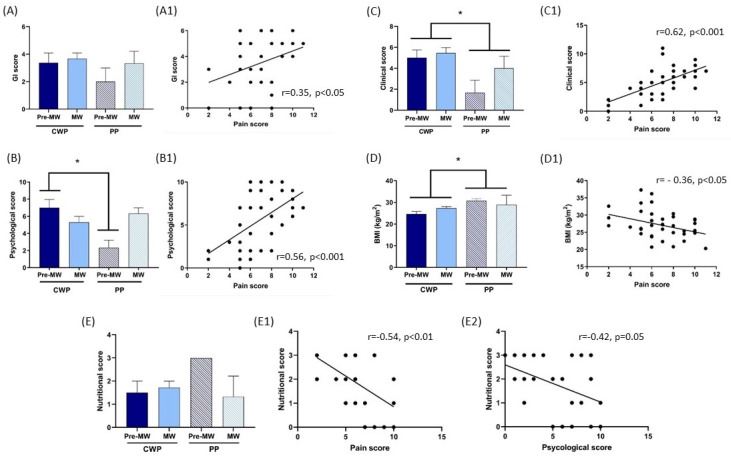
Gastrointestinal (GI) (**A**,**A1**), psychological (**B**,**B1**), clinical (**C**,**C1**), BMI values (**D**,**D1**) and nutritional score (**E**,**E1**,**E2**). Women were divided into four groups depending on the menopausal condition (reproductive period, pre-MW, *n* = 11; menopausal period, MW, *n* = 27) and type of pain (chronic widespread pain, CWP, *n* = 32; pelvic pain, PP, *n* = 6). Data are reported as mean ± SEM. Pearson r analysis for correlations. * *p* < 0.05 vs. same group/groups other pain.

**Gastrointestinal (GI) scores.** As reported in detail in Table 2 and Table S3, 32/38 (84%) women reported various types of disorders related to the gut. In particular, abdominal pain was reported by 26/38 (68%), abdominal swelling by 27/38 (71%) and gastroesophageal reflux by 23/38 (61%). Colitis was described by 23/38 women, while 10/38 reported constipation and 14/38 hemorrhoids. In 15 of them, 5 or 6 disorders per person were present. All these conditions were chronically present and were often treated with drugs. ANOVA applied to the GI scores with the factors menopause (pre-MW, MW) and pain (CWP, PP) revealed no significant differences among groups (Figure 3A); the GI score was positively correlated with the Pain score (*n* = 38, r = 0.35, *p* < 0.05) (Figure 3A1).

**Psychological score.** This score is composed of tests about general mental health (MCS of the SF-36), mood (the six subscales of POMS) and eating behavior disorders (EAT). As reported in Table 2, many women reported high scores in most of the parameters, supporting the presence of altered psychological conditions. Few women reported eating disorders. In particular, 24/38 women had high MCS (<50%, worst condition); 21/38 had high values in subscale T of POMS (>55, worst condition), 18/38 had high values in subscale D of POMS (>55, worst condition), 21/38 had high values in subscale A of POMS (>55, worst condition), 31/38 had low values in subscale V of POMS (<55, worst condition), 22/38 had high values in subscale S of POMS (>55, worst condition) and 18/38 had high values in subscale C of POMS (>55, worst condition); 8/35 women had high EAT values (>20, worst condition).

ANOVA applied to the psychological score (Figure 3B) with the factors menopause (pre-MW, MW) and pain (CWP, PP) revealed significance of the interaction between the two factors (F(1,34) = 3.93, *p* = 0.05) due to pre-MW/CWP having higher (worse) psychological scores than pre-MW/PP. The psychological score was positively correlated (Pearson correlation) with the pain score (*n* = 38, r = 0.56, *p* < 0.001), underlining the higher pain in women with worse psychological conditions (Figure 3B1).

**Clinical score.** Considering the various conditions used to calculate the clinical score, an endocrinopathy was present in 16/38 subjects, disturbed sleep in 26/38, at least one cardio-circulatory disorder in 25/38 and a urinary/kidney disorder in 24/38. ANOVA applied to the clinical score with the factors menopause (pre-MW, MW) and pain (CWP, PP) showed a significant difference (F(1,36) = 5.56, *p* = 0.05) due to this parameter being higher in CWP than in PP (Figure 3C). The clinical score was positively correlated (Pearson correlation) with the pain score (*n* = 38, r = 0.62, *p* < 0.001), highlighting that subjects with other pathologies showed the worst pain conditions (Figure 3C1).

**Reproductive score.** As shown in Table 2, also in this parameter, many women reported high scores, particularly related to the menstrual cycle. Indeed, 28/38 reported pain during the menstrual cycle, 24/38 a heavy menstrual cycle and 11/38 an irregular cycle; 8/38 had had at least one miscarriage; 27/38 were using or had used birth control pills; 11/27 menopausal women had encountered symptoms related to menopause. Only two of the menopausal women had taken hormones; 21/38 were suffering or had suffered from breast and ovarian-uterine disorders, 13/38 had never been pregnant. ANOVA applied to the reproductive score with the factors menopause (pre-MW, MW) and pain (CWP, PP) showed no significant differences (*p* > 0.05). No significant correlations were found between the reproductive score and pain score.

**Allergy score.** As reported in Table 2, 50% (19/38) of all the participants suffered from allergies (drug allergies, environmental allergies and/or food allergies). Food allergies were related to fruits and vegetables, nuts and eggs. The Kolmogorov-Smirnov test applied to the Allergy score with the factors Menopause (pre-MW, MW) and Pain (CWP, PP) showed no significant differences (*p* > 0.05). There was no significant correlation between the Allergy score and the Pain score.

**Drug score.** As reported in Table 2, 84% (32/38) of subjects were chronic drug users. Gastroprotective drugs were used by 11/38, cortisones by 4/38, opioids by 0/38, cannabinoids by 2/38, NSAIDs by 15/38, antipyretics by 12/38, antidepressants by 9/38, analgesics by 7/38, muscle relaxants by 5/38 and sedatives by 5/38. ANOVA applied to the drug score with the factors menopause (pre-MW, MW) and pain (CWP, PP) showed no significant differences (*p* > 0.05). The drug score was positively correlated with the pain score (*n* = 38, r = 0.53, *p* < 0.001), underlining the higher use of drugs in those with worse pain conditions.

**Physical activity score.** Only 2/38 subjects reported doing vigorous physical activity, while 19/38 did moderate physical activity and 13/38 walked at least 30 min a week (Table 2); 4/38 did not exercise. ANOVA applied to the physical activity score with the factors menopause (pre-MW, MW) and pain (CWP, PP) showed no significant differences (*p* > 0.05). There was no significant correlation between the physical activity score and the pain score.

**Body Mass Index (BMI).** Eleven women were in the normal weight range (BMI 18–25), while 27/38 had a BMI over 25, i.e., 16/27 were in the overweight category (BMI 25–30) and 11/27 were classified as obese (BMI 30+). ANOVA applied to BMI with the factors pain (CWP, PP) and menopause (pre-MW, MW) showed a significant difference in pain (F(1,34) = 4.72, *p* < 0.05) due to BMI being higher in the PP group than in CWP (Figure 3D). There was no significance for menopause or the interaction between the two factors (*p* > 0.05) (Figure 7(A)). BMI was negatively correlated with the pain score (*n* = 38, r = −0.36, *p* < 0.05), underlining the higher pain in women with lower BMI (Figure 3D1).

**Nutritional score.** The nutritional score considered data related to the BMI and selected BIA parameters in the 26 women in which the BIA was carried out (Table 2). All participants had fat-free mass (FFM) values in the reference range; 15/26 had high fat mass (FM), 3/26 had a low body cell mass index (BCMI) and only in 2/26 women was the total body water (TBW) lower than the reference value. ANOVA applied to the nutritional score with the factors menopause (pre-MW, MW) and pain (CWP, PP) showed no significant differences (*p* > 0.05) (Figure 3E). The nutritional score (whose higher values mean worse conditions) was negatively correlated with the pain score (*n* = 26, r = −0.54, *p* < 0.01) and psychological score (*n* = 26, r = −0.42, *p* = 0.05), highlighting the higher pain and worse psychological condition in women with a lower (better) nutritional score (Figure 3E1,3E2).

**Figure 4:**
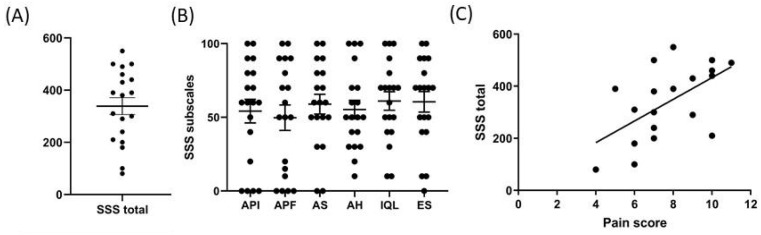
Gastrointestinal (GI) Symptom Severity Scale (SSS). (**A**) SSS total; (**B**) SSS subscales: abdominal pain intensity, API; abdominal pain frequency, APF; abdominal swelling, AS; abdominal heaviness, AH; interference with quality of life, IQL; evacuation satisfaction, ES. Higher values mean worse conditions. Data are reported as mean ± SEM, *n* = 19. (**C**) Pearson r analysis (r = 0.57, *p* < 0.01, *n* = 19).

**Gastrointestinal (GI) SSS.** The SSS questionnaire was completed by 19 subjects, all of whom suffered from CWP. The SSS total was 338.95 ± 32.41 (Table 2, Figure 4). In particular, 1/19 was in the normal SSS total range (< 90), while 3/19 had mild SSS total (90–210), 5/19 had a moderate SSS total (210–360) and 10/19 had severe SSS total (> 360). The six subscales were also analyzed individually: abdominal pain intensity (API) had a value of 54.21 ± 7.99, abdominal pain frequency (APF) 49.74 ± 8.60, abdominal swelling (AS) 58.95 ± 6.71, abdominal heaviness (AH) 55.26 ± 6.28, evacuation satisfaction (ES) 60.52 ± 6.94 and interference with quality of life (IQL) 61.05 ± 6.30. In the SSS subscales, severe cases were indicated by scores higher than 60; in particular, 8/19 women reported API, APF and AS values higher than 60, 6/19 women reported AH values higher than 60, 10/19 women reported IQL values higher than 60, 11/19 women reported ES values higher than 60. ANOVA applied to SSS (total or single scales) determined in pre-MW and MW showed no significant differences. The SSS total, determined in each subject, was positively correlated with the pain score (*n* = 19, r = 0.57, *p* < 0.01). The analysis per subscale showed that API, APF and AH were highly correlated with the pain score (respectively: r = 0.55, *p* < 0.01; r = 0.60, *p* < 0.01; r = 0.56, *p* < 0.01) (Figure S1).

**Figure 5:**
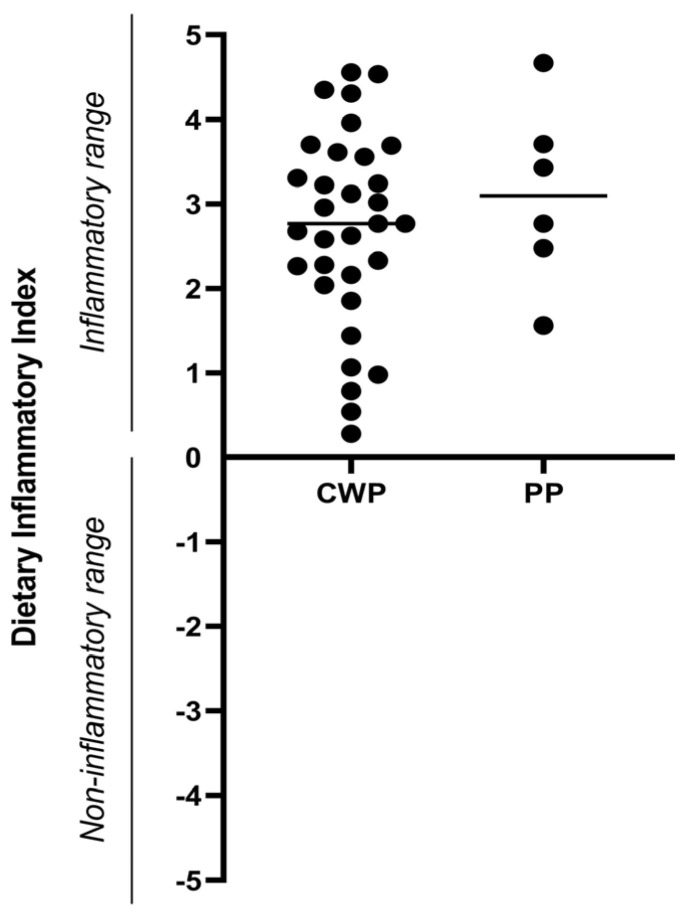
Dietary Inflammatory Index (DII). Women were divided into two groups depending on the type of pain (chronic widespread pain, CWP, *n* = 32; pelvic pain, PP, *n* = 6). The DII is considered noninflammatory in the range −5.5/0 or inflammatory (0/+5.5). Data are reported as single values and as mean values.

**Dietary Inflammatory Index (DII).** The DII was calculated from the 24HR feeding data only during Visit 1; it was not carried out at Visit 2 since all subjects had been asked to follow an anti-inflammatory diet between visits. As shown in Figure 5, the DII values were higher than 0 in all subjects, i.e., in the proinflammatory range; indeed, more than 50% of subjects showed very high values (>3). ANOVA applied to the DII score with the factors menopause (pre-MW, MW) and pain (CWP, PP) showed no significant differences (*p* > 0.05). No significant correlation was found between the DII and the pain score. In fact, the DII was very high in all subjects independently of age and pain levels, indicating the presence of foods able to induce inflammation in the meals of all subjects.

**Personalized diet.** Each subject’s feeding habits were recorded and discussed in detail by a team of nutritionists, and the best diet was chosen based generally on the Mediterranean diet but fully personalized according to the patient’s health features. Of the 38 participants, 13 suffered from food intolerances. In particular, 9/38 reported intolerance to dairy products, although only 3 of these subjects did not consume milk. On the basis of intolerances and personal choices related to their health, 26/38 had already reduced some foods in their diet, especially some types of cereals and foods containing gluten and milk and its derivatives. In all cases, the exclusion was not total.

Seventeen different diet variations were suggested, with a number of differences one from another. The list of suggestions was prepared independently of the patient’s agreement to follow it and/or to participate in the second visit.

Out of the 38 subjects, 26 followed the indications and attended the follow-up visit (Visit 2).

**Figure 6:**
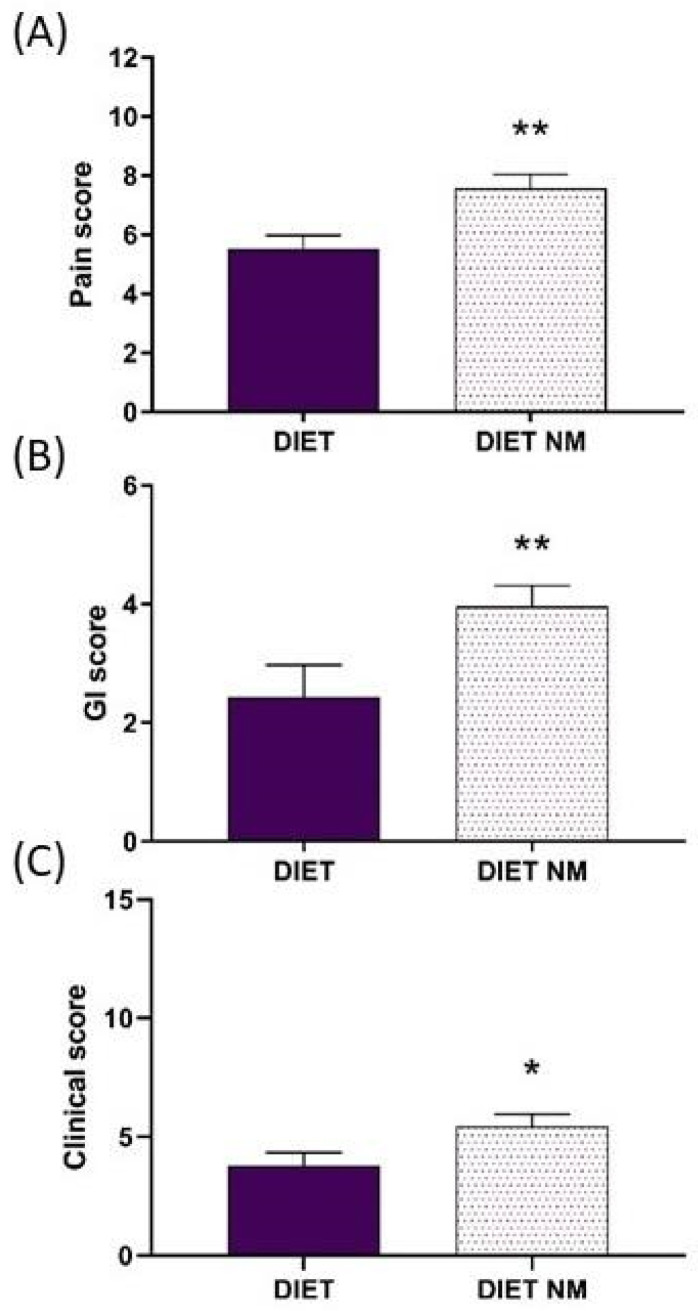
Pain (**A**), gastrointestinal (GI, **B**) and clinical (**C**) scores determined in the two groups (diet with milk, DIET, *n* = 7 and diet without milk, DIET NM, *n* = 19) during the first visit (Visit 1). * *p* < 0.05, ** *p* < 0.01 vs. DIET group. Data are reported as mean ± SEM.

To better point out the role of dairy products in pain and other parameters, a further analysis was carried out on data collected during Visit 1 by dividing the subjects into two groups depending on the presence of milk and other dairy products in the food list received, i.e., factor milk (2 levels: diet with milk, DIET, *n* = 14; diet without milk, DIET NM, *n* = 23). One-way ANOVA applied to the calculated scores with the factor milk showed a significant difference between the two groups for the pain score (F(1,35) = 8.37, *p* < 0.01), clinical score (F(1,35) = 4.41, *p* < 0.05) and GI score (F(1,35) = 6.11, *p* < 0.01), due in all cases to higher values (worse condition) in subjects belonging to the DIET NM group (Figure 6).

**Comparisons between the two visits.** In the 26 women for whom a follow-up was carried out after 4 weeks, an ANOVA was used to compare the different scores between the first and second visits with the factors Test (Visit 1 and Visit 2) and Milk (DIET and DIET NM). Only significant results are reported, independently of the group.

**Figure 7:**
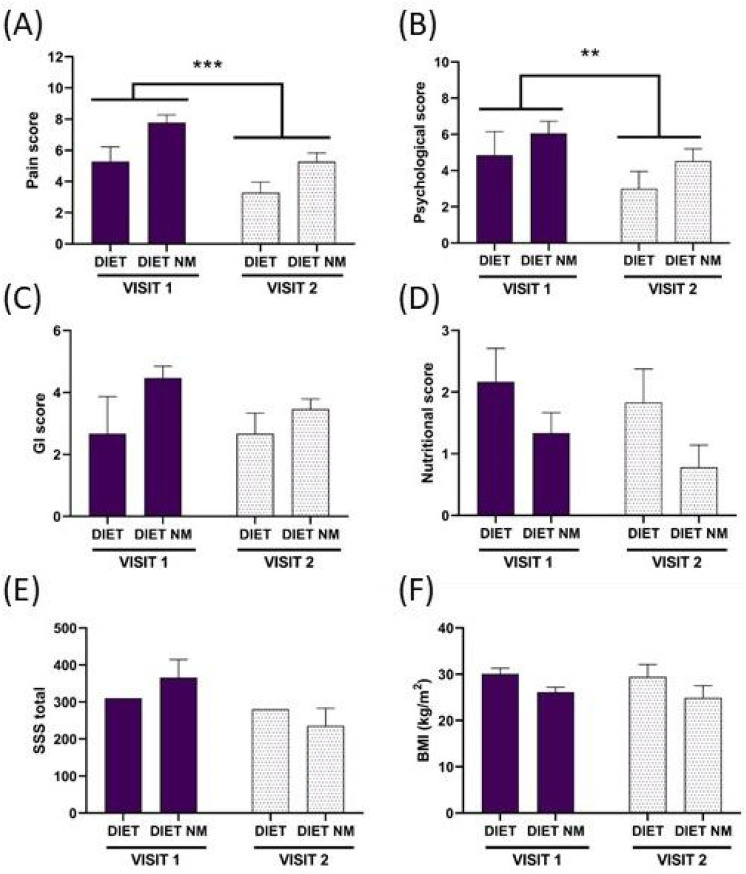
Pain (**A**), Psychological (**B**), gastrointestinal (GI, **C**), nutritional (**D**) scores, SSS total (**E**) and BMI (**F**) determined in the two groups (diet with milk, DIET, *n* = 7 and diet without milk, DIET NM, *n* = 19) during the first visit (Visit 1) and follow-up visit (Visit 2). Data are reported as mean ± SEM. ** *p* < 0.01 vs. same groups Visit 1; *** *p* < 0.001 vs. same groups Visit 1.

Pain score (Figure 7). Repeated measures ANOVA applied to the pain score with the factors test and milk showed significant differences for both factors (F(1,24) = 29.68, *p* < 0.001, F(1,24) = 6.12, *p* < 0.05, respectively) due to the higher pain score in the DIET NM group than in the DIET one and the lower pain score at Visit 2 than at Visit 1 independent of group.

Psychological score (Figure 7). Repeated measures ANOVA showed significance of the factor test (F(1,24) = 9.03, *p* < 0.01, *n* = 26) due to the lower score at Visit 2 than at Visit 1.

## 5. Discussion

The main results of the present study can be summarized in two points: the first is related to the several pieces of evidence suggesting strong interactions between the chronic pain condition suffered by each subject and the subjects’ gastrointestinal health; the second underlines the ability of a personalized diet to significantly improve the chronic pain condition.

Chronic pain is multifaceted and can originate from several conditions mixed in thousands of combinations. The clinical result is unique; indeed, chronic pain represents the essence of the need to create personalized medicine. Each subject has his/her personal, familial and social history. Age and sex are likewise fundamental [26,27] and food can be added to this list of factors [28]. Of particular interest among the various efforts to find causes/therapeutics to fight pain is the recent attention towards possible interactions between gastrointestinal (GI) health and chronic pain conditions. Several studies have been carried out to determine the potential benefits of foods considered to be anti-inflammatory [1,2,9,29]. Moreover, the modulatory role of the microbiota has been studied [30] and several gut-related biomarkers have been determined in the blood [31,32]. All these studies have suggested a beneficial effect of specific attention to the gut and the foods commonly consumed by pain patients. A list of potentially pro- or anti-inflammatory foods is available, and thus diets are provided that include known anti-inflammatory foods and/or eliminate known inflammatory ones [33].

The present results clearly indicate that chronic pain can be related to the GI conditions and that one month of feeding only with anti-inflammatory foods is able to significantly improve pain and other important aspects of a patient’s quality of life. In this sample, independent of the nature of the pain, more than 80% of subjects reported GI disorders such as gastroesophageal reflux, diarrhea and/or constipation, abdominal swelling and colitis; such conditions are very common because the gut is highly responsive to many internal and/or external factors able to disrupt its physiological functions. In chronic pain patients, there is a high probability of the chronic use of analgesics. This class of drugs, independent of the strength and dosage, always affects the gut, notwithstanding the huge efforts carried out by physicians to fight side effects. The inhibitory action of opioids on GI transit is well known, constipation being a common side effect of opioids; but less considered is the fact that opioids inhibit GI secretions with less mucus and thus fewer digestive enzymes [34]. Importantly, also NSAIDs, antacids and antidepressants (able to disrupt 5-HT metabolism) have important effects on the GI tract [35,36]. Therefore, due to their long-term use, it can be hypothesized that the vicious cycle once started would never stop.

Foods are the other big variable able to affect GI functions. In the present study, we confirmed that foods used by the subjects in their meals were part of the problem since ALL of the subjects had values of the Dietary Inflammatory Index > 0, i.e., in the inflammatory range; thus, they were eating foods able to inflame the gut. In the present group of women, most were intolerant to dairy products, even though many of them continued to include these products in their diet. The continuing consumption of foods (like milk) not compatible with the subject’s gut can induce low-grade inflammation [37], which can be initiated or maintained even though a clear pathology is not necessarily manifested [38]. An important consequence of this condition is that the gut barrier integrity can be affected, with cellular alterations able to disrupt the regular digestion/absorption of foods/nutrients [38,39]. Two main consequences have to be considered: the first is the lack of an adequate intake of nutrients, for instance Vit. D, Vit. B12, etc.; the second is that the poor digestion of foods will allow the arrival of undigested elements in the colon, able to significantly alter the gut microbiota with long-lasting effects (i.e., decrease in short-chain fatty acids, SCFAs) and to produce products (i.e., gas) able to give other clinical consequences [40]. These effects were summarized by the GI SSS test, in which most of the women showed very high values, indicating discomfort.

In the women intolerant of dairy products, it was suggested to completely avoid them. One reason to adopt this solution is suggested by the presence in these products of advanced glycation end products (AGEs) and advanced lipoxidation end products (ALEs), substances resulting from nonenzymatic modifications of proteins potentially harmful to human health [41]. These substances are associated with activation of pro-inflammatory transcription nuclear factor kappa-light-chain-enhancer of activated B cells (NFkB) [42]. Reducing the tissue accumulation of AGE and ALE ligands has been shown to improve inflammation [43]. Interestingly, in our sample of women, the pain scores were higher in those reporting allergies/intolerances to dairy products, and a diet without these foods significantly decreased the pain.

In conclusion, in the present study, the detailed description of many parameters with particular attention to the gastrointestinal tract suggests that, in women with chronic pain, the gut can significantly be affected by foods and by drugs, things apparently good for health but with many dark sides to be considered. In particular, in pain patients, allergies/intolerances to foods like dairy products must be carefully ascertained to avoid food-induced inflammation and then higher drug-resistant pain.

## Figures and Tables

**Table 1:** **Demographic data.** Women were divided into two groups depending on the menopausal condition (pre-MW: premenopausal women; MW: menopausal women). Abbreviations: chronic widespread pain (CWP); pelvic pain (PP).

Groups	Number	Age	Pain
Pre-MW	11	34.18	CWP = 8
		(±2.74)	PP = 3
MW	27	60.04 (±1.23)	CWP = 24 PP = 3

**Table 2:** All scores. Detailed list of scores calculated per subject during the first visit (V1) and follow-up visit (V2). Abbreviations: visit in person (P), visit online (O), premenopausal women (pre-MW), menopausal women (MW), gastrointestinal (GI), body mass index (BMI). na: data not available.

Cod Sub	P/O	Age	Pre-MW MW	BMI V1–V2	Pain Scores V1–V2	GI Scores V1–V2	Psycho Scores V1–V2	Clinical Scores	Reprod Scores	Allergy Scores	Drugs Scores	Activity Scores V1–V2	Nutri Scores V1–V2	Diet Sugg Yes/No
1	P	26	PRE-MW	29–na	2–na	3–na	2–na	0	5	2	1	1–na	3–na	No
2	P	50	PRE-MW	30–na	8–na	0–na	4–na	4	3	0	4	2–na	3–na	No
3	P	60	MW	22–23	9–5	5–na	5–2	6	3	0	1	1–1	0–0	Yes
4	P	31	PRE-MW	32–32	2–1	3–na	1–1	1	4	1	0	1–2	3–3	Yes
5	P	59	MW	37–36	5–3	3–na	7–3	4	3	0	0	2–3	3–3	Yes
6	P	67	MW	27–na	6–na	2–na	7–na	2	2	0	2	2–na	1–na	No
7	P	56	MW	36–34	6–3	0–na	9–4	5	2	0	0	2–2	3–3	Yes
8	P	60	MW	26–na	5–na	0–na	2–na	1	1	0	1	1–na	2–na	No
9	P	55	MW	31–na	5–2	4–na	0–0	3	3	1	1	2–na	3–na	Yes
10	P	37	PRE-MW	28–na	6–na	2–na	9–na	2	3	0	1	2–na	2–na	No
11	P	49	MW	26–na	2–1	0–na	1–0	2	2	0	0	2–na	2–na	Yes
12	P	57	MW	22–na	7–4	3–na	7–2	2	5	1	1	1–2	0–na	Yes
13	P	72	MW	24–23	5–3	5–na	5–4	5	3	0	2	1–na	2–1	Yes
14	P	19	PRE-MW	20–21	6–9	0–na	10–8	3	4	0	0	0–na	1–2	Yes
15	P	56	MW	25–na	5–na	6–na	3–na	5	7	1	1	1–na	3–na	No
16	P	54	MW	33–32	6–3	5–na	4–1	5	5	1	1	1–na	3–3	Yes
17	P	61	MW	29–28	8–5	1–2	8–7	6	3	1	2	2–na	3–1	Yes
18	P	69	MW	27–23	10–5	5–4	8–4	4	5	0	2	1–na	1–0	Yes
19	P	57	MW	20–20	8–5	2–2	6–5	5	5	2	2	2–1	0–0	Yes
20	O	26	PRE-MW	27–na	7–na	2–na	9–na	6	2	3	2	1–na	na–na	No
21	P	62	MW	28–na	7–5	6–5	9–2	11	3	2	3	3–na	na–na	Yes
22	O	64	MW	25–na	9–na	4–na	10–na	7	4	1	2	2–na	0–na	No
23	P	39	PRE-MW	23–na	6–2	3–3	4–0	5	4	0	3	3–3	na–na	Yes
24	P	68	MW	23–23	6–5	5–4	2–1	7	7	0	4	1–1	1–1	Yes
25	P	73	MW	24–23	10–8	4–3	9–6	9	4	1	2	1–na	0–0	Yes
26	P	67	MW	28–28	10–7	4–2	6–6	7	4	0	2	1–na	2–1	Yes
27	O	47	PRE-MW	20–na	11–5	5–4	7–6	7	6	0	2	2–2	na–na	Yes
28	O	62	MW	24–na	9–7	5–3	9–9	6	5	0	4	1–na	na–na	Yes
29	O	35	PRE-MW	28–na	10–10	6–5	7–7	8	5	2	2	1–na	na–na	Yes
30	O	34	PRE-MW	22–na	7–na	4–na	2–na	3	4	3	3	2–na	na–na	No
31	O	32	PRE-MW	25–na	10–8	5–4	8–8	6	5	4	4	1–na	na–na	Yes
32	P	57	MW	26–25	4–4	2–1	3–1	4	5	3	2	2–2	2–0	Yes
33	O	57	MW	26–na	8–4	5–3	2–6	7	3	2	2	2–2	na–na	Yes
34	O	57	MW	24–na	8–7	6–5	10–8	8	3	1	2	2–na	na–na	Yes
35	O	46	MW	27–na	7–na	6–na	10–na	10	5	0	3	3–na	na–na	No
36	P	55	MW	25–22	7–2	2–3	2–6	5	5	2	2	2–2	1–0	Yes
37	P	61	MW	33–na	5–na	6–na	1–na	5	4	1	na	na–na	na–na	No
38	P	60	MW	30–na	6–na	2–na	1–na	2	5	0	na	na–na	3–na	No

## Data Availability

The data are available from the authors upon reasonable request.

## Supplementary Materials

The following supporting information can be downloaded at: https://www.mdpi.com/article/10.3390/ijerph21111435/s1, Figure S1: Correlation of Symptom Severity Scale (SSS) subscales and Pain score. Correlations: A) abdominal pain intensity, API; B) abdominal pain frequency, APF; C) abdominal heaviness, AH. Pearson r correlation (respectively: r = 0.55, r = 0.60, r = 0.56, all *p* < 0.01, N = 19); Table S1: Score construction table. Pain score: four components of the Pain score: Visual Analogue Scale (VAS) mean of three parts of the day (morning, afternoon and night) (m3VAS), Margolis (MA) questionnaire mean of three parts of the day (morning, afternoon and night) (m3MA), Pain Rating Index rank-Total (PRIr-T) of the Italian Pain Questionnaire (QUID), Bodily Pain (BP) of the Short Form-36 questionnaire (SF-36) (in this scale, higher values indicate a better status). Each component was divided into four ranges. Each range corresponds to a score (0, best condition—3, worst condition). Gastrointestinal (GI) score: six components of the GI score. In the presence of the conditions, the score was 1 (worst condition), in the absence of the conditions the score was 0 (best condition). Psychological score: eight components of the Psychological score: Mental Component Summary (MCS) of the SF-36, six subscales (Tension-Anxiety, T, Depression-Dejection, D, Anger-Hostility, A, Vigor-Activity, V, Fatigue-Inertia, S, Confusion-Bewilderment, C) of Profile of Mood States (POMS), and Eating Attitude Test (EAT). MCS values were divided into four ranges, each range corresponding to a score (0, best condition—3, worst condition). POMS subscales values and EAT values were divided into two ranges: inside (score 0, best condition) or outside (score 1, worst condition) the reference values. Clinical score: fifteen components of the Clinical score: the components were grouped into four types of disorders. In the presence of the conditions the score was 1 (worst condition), in the absence of the conditions the score was 0 (best condition). Reproductive score: ten components of the Reproductive score. The best condition was marked with score 0, the worst condition was marked with 1. Allergy score: three components of the Allergy score. In the presence of the conditions the score was 1 (worst condition), in the absence of the conditions the score was 0 (best condition). Drug score: components of the Drug score: ten drug classes. Chronic drug intake was marked with score 1 (worst condition), failure to take the drug was marked with 0 (best condition). Abbreviation: Nonsteroidal anti-inflammatory drugs (NSAIDs). Physical activity score: three intensity levels of physical activity—for each level the minimum value accepted was 30 min/week. If the minimum value was reached, the score was 0 (best condition), if the activity level was lower than the established minimum value the assigned score was 1 (worst condition). Nutritional score: Body Mass Index (BMI) and Body composition (fat free mass, FFM, fat mass, FM, body cell mass index, BCMI, and total body water, TBW). BMI values were divided into three ranges, each range corresponding to a score (0, best condition—2, worst condition). The parameters of body composition were divided into two ranges: inside (score 0, best condition) or outside (score 1, worst condition) the reference values, Table S2: Pain score. Detailed list of attributed scores per subject during first visit (Visit 1) and follow up visit (Visit 2). Abbreviations: Visual Analogue Scale (VAS), Margolis (MA) questionnaire, Pain Rating Index rank-Total (PRIr-T) of the Italian Pain Questionnaire (QUID), Bodily Pain (BP) scale present in the Short Form-36 (SF-36) questionnaire. Values for each parameter were subdivided into 4 scores (0, best condition—3, worst condition); the VAS and MA data reported in the table is the mean of the three daily determinations (morning, afternoon and night). n/a: if the subject was not present at the second visit, Table S3: Gastrointestinal (GI) score. Detailed list of attributed scores per subject. Each parameter was subdivided into 2 scores (No—no disorders, score 0; Yes—yes disorders, score 1).

## Abbreviations

24HR	24-h dietary recall
AGEs	advanced glycation end products
AH	abdominal heaviness
ALEs	advanced lipoxidation end products
ANOVA	analysis of variance
APF	abdominal pain frequency
API	abdominal pain intensity
AS	abdominal swelling
BCMI	body cell mass index
BIA	bioelectrical impedance analysis
BMI	body mass index
BP	bodily pain
CWP	chronic widespread pain
DIET NM	diet without milk
DIET	diet with milk
DII	Dietary Inflammatory Index
EAT	Eating Attitude Test
ES	evacuation satisfaction
FFM	fat free mass
FM	fat mass
FODMAP	fermentable oligosaccharides, disaccharides, monosaccharides and polyols
GI	Gastrointestinal
IBS	irritable bowel syndrome
IgG4	immunoglobulin G4
IPAQ	international physical activity questionnaire
IQL	interference with quality of life
LSD	least significant difference
MA	Margolis
MCS	Mental Component Summary: vitality (V), social functioning (SF), emotional role (ER), mental health (MH)
MW	menopausal women
NFkB	nuclear factor kappa-light-chain-enhancer of activated B cells
NSAIDs	nonsteroidal anti-inflammatory drugs
POMS	Profile of Mood States: Tension-Anxiety (T), Depression-Dejection (D), Anger-Hostility (A), Vigor-Activity (V), Fatigue-Inertia (F), Confusion-Bewilderment (C)
PP	pelvic pain
Pre-MW	premenopausal women
PRIr-T	Pain Rating Index rank-Total
PUFA	polyunsaturated fatty acid
QUID	Italian Pain Questionnaire: sensory (S), affective (A), emotional (E), miscellaneous (M)
SEM	standard error of the mean
SF-36	Short Form-36
SSS	symptom severity scale
TBW	total body water
VAS	visual analogue scale
