# Supplementary material for: Gastrointestinal Conditions Affect Chronic Pain and Quality of Life in Women
Source: Int J Environ Res Public Health. 2024 Oct 29;21(11):1435. doi: 10.3390/ijerph21111435 (PMC11593551; doi:10.3390/ijerph21111435)
Supplement: Supplementary file 1 [file ijerph-21-01435-s001.zip › ijerph-3240439-supplementary.pdf]

Supplementary figures:

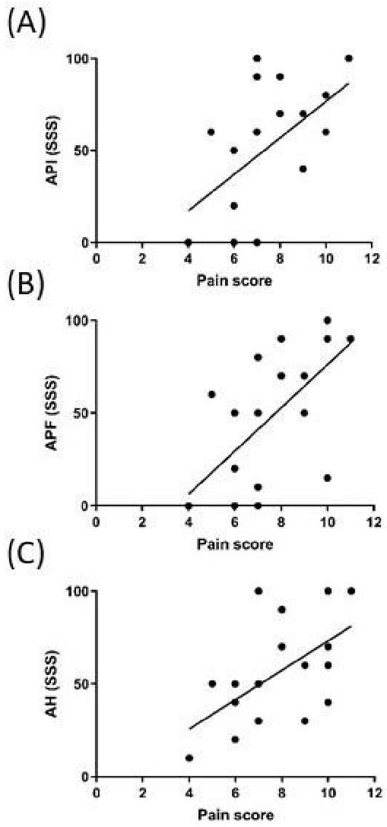

Figure S1 supp. **Correlation of Symptom Severity Scale (SSS) subscales and Pain score.** Correlations of a. abdominal pain intensity, API; b. abdominal pain frequency, APF; c. abdominal heaviness, AH with Pain score. Pearson r analysis was used to determine the significance of the correlation (respectively: r=0.55, r=0.60, r=0.56, all p<0.01, N=19).

Supplementary tables:

Table S1 supp. **Score construction table. Pain score:** four components of the Pain score: Visual Analogue Scale (VAS) mean of three parts of the day (morning, afternoon and night) (m3VAS), Margolis (MA) questionnaire mean of three parts of the day (morning, afternoon and night) (m3MA), Pain Rating Index rank-Total (PRIr-T) of the Italian Pain Questionnaire (QUID), Body Pain (BP) of the Short Form-36 questionnaire (SF-36) (in this scale, higher values indicate a better status). Each component was divided into four ranges. Each range corresponds to a score (0, best condition – 3, worst condition). **Gastrointestinal (GI) score:** six components of the GI score. In the presence of the conditions, the score was 1 (worst condition), in the absence of the conditions the score was 0 (best condition). **Psychological score:** eight components of the Psychological score: Mental Component Summary (MCS) of the SF-36, six subscales (Tension-Anxiety, T, Depression-Dejection, D, Anger-Hostility, A, Vigor-Activity, V, Fatigue-Inertia, S, Confusion-Bewilderment, C) of Profile of Mood States (POMS), and Eating Attitude Test (EAT). MCS values were divided into four ranges, each range corresponding to a score (0, best condition – 3, worst condition). POMS subscales values and EAT values were divided into two ranges: inside (score 0, best condition) or outside (score 1, worst condition) the reference values. **Clinical score:** fifteen components of the Clinical score: the components were grouped into four types of disorders. In the presence of the conditions the score was 1 (worst condition), in the absence of the conditions the score was 0 (best condition). **Reproductive score:** ten components of the Reproductive score. The best condition was marked with score 0, the worst condition was marked with 1. **Allergy score**: three components of the Allergy score. In the presence of the conditions the score was 1 (worst condition), in the absence of the conditions the score was 0 (best condition). **Drug score:** components of the Drug score: ten drug classes. Chronic drug intake was marked with score 1 (worst condition), failure to take the drug was marked with 0 (best condition). Abbreviation:

Nonsteroidal anti-inflammatory drugs (NSAIDs). **Physical activity score:** three intensity levels of physical activity - for each level the minimum value accepted was 30 minutes/week. If the minimum value was reached, the score was 0 (best condition), if the activity level was lower than the established minimum value the assigned score was 1 (worst condition). **Nutritional score:** Body Mass Index (BMI) and Body composition (fat free mass, FFM, fat mass, FM, body cell mass index, BCMI, and total body water, TBW). BMI values were divided into three ranges, each range corresponding to a score (0, best condition – 2, worst condition). The parameters of body composition were divided into two ranges: inside (score 0, best condition) or outside (score 1, worst condition) the reference values.

	**0**	**1**	**2**	**3**
**Pain score (0-12)**				
m3VAS	0-2.5	3-5.5	6-8	8.5-10
m3MA	0-5	5.5-10	10.5-25	>25
PRIr-T (QUID)	0-10	10.5-20	20.5-30	>30
BP (SF-36)	>60	40-60	20-40	40-60

**GI score (0-6)**				
Dry mouth	No	Yes		
Gastroesophageal reflux	No	Yes		
Abdominal pain	No	Yes		
Abdominal swelling	No	Yes		
Colitis	No	Yes		
Hemorrhoids	No	Yes		
**Psychological score (0-10)**				
MCS (SF-36)	>60	40-60	20-40	0-20
POMS T	≤55	>55		
POMS D	≤55	>55		

POMS A	≤55	>55		
POMS V	>55	≤55		
POMS S	≤55	>55		
POMS C	≤55	>55		
EAT	<20	≥20		
**Clinical score (0-15)**				
Endocrinopathies: Thyroid	No	Yes		
Endocrinopathies: Diabetes	No	Yes		
Sleep quality	No	Yes		
Insomnia	No	Yes		
Frequent awakenings	No	Yes		

Snoring	No	Yes		
Lymphatic disorders	No	Yes		
Vascular disorders	No	Yes		
Cardiac disorders	No	Yes		
Anemia	No	Yes		
Blood pressure	No	Yes		
Cholesterol	No	Yes		
Kidney	No	Yes		
Bladder	No	Yes		
Candida	No	Yes		
**Reproductive score (0-10)**				

Painful menstrual cycle	No	Yes		
Heavy menstrual cycle	No	Yes		
Irregular menstrual cycle	No	Yes		
Spontaneous abortions	No	Yes		
Pregnancies	No	Yes		
Contraceptives	No	Yes		
Menopause symptoms	No	Yes		
Hormonal therapy	No	Yes		
Breast	No	Yes		
Ovarian-uterine pathologies	No	Yes		
**Allergies (0-3)**				

Drug allergies	No	Yes		
Environmental allergies	No	Yes		
Food allergies	No	Yes		
**Drug score (0-10)**				
Gastroprotective products	No	Yes		
Cortisones	No	Yes		
Opioids	No	Yes		
Cannabinoids	No	Yes		
NSAIDs	No	Yes		
Antipyretics	No	Yes		
Antidepressants	No	Yes		

Analgesics	No	Yes		
Muscle relaxants	No	Yes		
Sedatives	No	Yes		
**Physical activity score (0-3)**	No	Yes		
Vigorous physical activity	No	Yes		
Moderate physical activity	No	Yes		
Walking activity	No	Yes		
**Nutritional score (0-6)**				
BMI	18-25	25-30	30+	
FFM	≥ 36.6	<36.6		
FM	≤ 22.6	>22.6		

BCMI	≥ 8	<8		
TBW	Pre-MW: ≥35L<br>MW: ≥26L	Pre-MW: <35L<br>MW: <26L		

Table S2 supp. **Pain score.** Detailed list of attributed scores per subject during first visit (Visit 1) and follow up visit (Visit 2). Abbreviations: Visual Analogue Scale (VAS), Margolis (MA) questionnaire, Pain Rating Index rank-Total (PRIr-T) of the Italian Pain Questionnaire (QUID), Bodily Pain (BP) scale present in the Short Form-36 (SF-36) questionnaire.Values for each parameter were subdivided in 4 scores (0, best condition – 3, worse condition); the VAS and MA data reported in the table is the mean of the three daily determinations (morning, afternoon and night). n/a: if the subject was not present at the second visit.

	**VAS mean (score 0-3)**		**MA mean (score 0-3)**		**PRIr-T (QUID) (score 0-3)**		**BP (SF-36) (score 0-3)**	
	Visit 1	***Visit 2***	***Visit 1***	***Visit 2***	***Visit 1***	***Visit 2***	***Visit 1***	***Visit 2***
**Sucject 1**	1 (0)	n/a	2,7 (0)	n/a	4 (0)	n/a	77,5 (0)	n/a
**Sucject 2**	7 (2)	n/a	7,0 (1)	n/a	43 (3)	n/a	22,5 (2)	n/a
**Sucject 3**	6 (2)	4 (1)	14,5 (2)	14,5 (2)	48 (3)	20 (1)	35,0 (2)	57,5 (1)
**Sucject 4**	5 (1)	4 (1)	7,6 (1)	7,6 (1)	25 (2)	9 (0)	22,5 (2)	45,0 (1)
**Sucject 5**	3 (3)	1 (0)	9,5 (1)	9,5 (1)	6 (0)	5 (0)	67,5 (0)	67,5 (0)
**Sucject 6**	6 (2)	5 (1)	9,0 (1)	6,0 (1)	10 (0)	5 (0)	45,0 (1)	45,0 (1)
**Sucject 7**	5 (1)	n/a	7,0 (1)	n/a	51 (3)	n/a	47,5 (1)	n/a
**Sucject 8**	6 (2)	n/a	9,0 (1)	n/a	5 (0)	n/a	22,5 (2)	n/a

**Sucject 9**	8 (2)	4 (1)	4,0 (0)	4,0 (0)	3 (0)	11 (1)	12,5 (3)	80,0 (0)
**Sucject 10**	7 (2)	n/a	20,7 (2)	n/a	13 (1)	n/a	45,0 (1)	n/a
**Sucject 11**	2 (0)	1 (0)	4,0 (0)	4,0 (0)	9 (0)	14 (1)	35,0 (2)	90,0 (0)
**Sucject 12**	6 (2)	3 (1)	11,5 (2)	19,5 (2)	13 (1)	6 (0)	22,5 (2)	55,0 (1)
**Sucject 13**	8 (2)	2 (0)	5,0 (0)	9,8 (1)	6 (0)	n/a	33,3 (2)	25,0 (2)
**Sucject 14**	6 (2)	7 (2)	8,2 (1)	30,2 (3)	13 (1)	26 (2)	35,0 (2)	22,5 (2)
**Sucject 15**	5 (1)	n/a	11,7 (2)	n/a	13 (1)	n/a	55,0 (1)	n/a
**Sucject 16**	5 (1)	4 (1)	28,3 (3)	22,8 (2)	13 (1)	7 (0)	45,0 (1)	67,5 (0)
**Sucject 17**	8 (2)	4 (1)	60,0 (3)	32,0 (2)	16 (1)	6 (0)	22,5 (2)	45,0 (1)
**Sucject 18**	6 (2)	7 (2)	38,9 (3)	25,3 (3)	42 (3)	10 (0)	35,0 (2)	45,0 (1)
**Sucject 19**	7 (2)	5 (1)	54,3 (3)	35,5 (3)	18 (1)	10 (0)	32,5 (2)	45,0 (1)
**Sucject 20**	7 (2)	n/a	17,5 (2)	n/a	15 (1)	n/a	32,5 (2)	n/a

**Sucject 21**	7 (2)	7 (2)	21,0 (2)	18,2 (2)	15 (1)	5 (0)	22,5 (2)	45,0 (1)
**Sucject 22**	6 (2)	n/a	19,0 (2)	n/a	36 (3)	n/a	35,0 (2)	n/a
**Sucject 23**	8 (2)	1 (0)	18,7 (2)	14,0 (2)	18 (1)	1 (0)	45,0 (1)	90,0 (0)
**Sucject 24**	4 (1)	4 (1)	40,5 (3)	45,3 (3)	15 (1)	3 (0)	45,0 (1)	57,5 (1)
**Sucject 25**	7 (0)	n/a	48,3 (3)	98,0 (3)	61 (3)	74 (3)	22,5 (2)	22,5 (2)
**Sucject 26**	8 (2)	5 (1)	10,7 (2)	46,7 (3)	39 (3)	23 (2)	0,0 (3)	45,0 (1)
**Sucject 27**	8 (2)	6 (2)	37,9 (3)	23,3 (2)	37 (3)	15 (1)	12,5 (3)	70,0 (0)
**Sucject 28**	6 (2)	5 (1)	22,5 (2)	38,0 (3)	37 (3)	20 (1)	32,5 (2)	32,5 (2)
**Sucject 29**	6 (2)	7 (2)	41,0 (3)	43,5 (3)	36 (3)	53 (3)	35,0 (2)	35,0 (2)
**Sucject 30**	5 (1)	n/a	35,0 (3)	n/a	23 (2)	n/a	45,0 (1)	n/a
**Sucject 31**	7 (2)	7 (2)	31,1 (3)	31,1 (3)	31 (3)	11 (1)	35,0 (2)	35,0 (2)
**Sucject 32**	5 (1)	3 (1)	14,5 (2)	27,0 (3)	9 (0)	7 (0)	45,0 (1)	67,5 (0)

**Sucject 33**	5 (1)	4 (1)	41,0 (3)	13,0 (2)	22 (2)	6 (0)	32,5 (2)	45,0 (1)
**Sucject 34**	8 (2)	8 (2)	24,4 (2)	42,2 (3)	11 (1)	10 (0)	0,0 (3)	22,5 (2)
**Sucject 35**	3 (1)	n/a	11,0 (2)	n/a	21 (2)	n/a	22,5 (2)	n/a
**Sucject 36**	4 (1)	1 (0)	18,0 (2)	24,2 (2)	21 (2)	2 (0)	32,5 (2)	67,5 (0)
**Sucject 37**	3 (1)	n/a	8,7 (1)	n/a	24 (2)	n/a	45,0 (1)	n/a
**Sucject 38**	6 (2)	n/a	7,3 (1)	n/a	20 (1)	n/a	45,0 (1)	n/a

Table 3 supp. **Gastrointestinal (GI) score.** Detailed list of attributed scores per subject. Each parameter was subdivided in 2 scores (No- no disorders, score 0; Yes- yes disorders, score 1).

	**Dry mouth (score 0-1)**	**Gastroesophageal reflux (score 0-1)**	**Abdominal pain (score 0-1)**	**Abdominal swelling (score 0-1)**	**Colitis (score 0-1)**	**Hemorrhoids (score 0-1)**
**Sucject 1**	No (0)	Yes (1)	Yes (1)	Yes (1)	No (0)	No (0)
**Sucject 2**	No (0)	No (0)	No (0)	No (0)	No (0)	No (0)
**Sucject 3**	No (0)	Yes (1)	Yes (1)	Yes (1)	Yes (1)	Yes (1)
**Sucject 4**	No (0)	No (0)	No (0)	No (0)	No (0)	No (0)
**Sucject 5**	No (0)	No (0)	Yes (1)	Yes (1)	Yes (1)	No (0)
**Sucject 6**	No (0)	Yes (1)	Yes (1)	Yes (1)	No (0)	No (0)
**Sucject 7**	Yes (1)	No (0)	No (0)	No (0)	No (0)	Yes (1)
**Sucject 8**	No (0)	No (0)	No (0)	No (0)	No (0)	No (0)
**Sucject 9**	No (0)	Yes (1)	Yes (1)	Yes (1)	Yes (1)	No (0)

**Sucject 10**	No (0)	No (0)	Yes (1)	Yes (1)	No (0)	No (0)
**Sucject 11**	No (0)	No (0)	No (0)	No (0)	No (0)	No (0)
**Sucject 12**	No (0)	No (0)	Yes (1)	Yes (1)	Yes (1)	No (0)
**Sucject 13**	Yes (1)	Yes (1)	Yes (1)	Yes (1)	Yes (1)	No (0)
**Sucject 14**	No (0)	No (0)	No (0)	No (0)	No (0)	No (0)
**Sucject 15**	Yes (1)	Yes (1)	Yes (1)	Yes (1)	Yes (1)	Yes (1)
**Sucject 16**	Yes (1)	Yes (1)	Yes (1)	Yes (1)	Yes (1)	No (0)
**Sucject 17**	Yes (1)	No (0)	No (0)	No (0)	No (0)	No (0)
**Sucject 18**	Yes (1)	Yes (1)	Yes (1)	Yes (1)	Yes (1)	No (0)
**Sucject 19**	Yes (1)	Yes (1)	No (0)	No (0)	No (0)	No (0)
**Sucject 20**	No (0)	No (0)	Yes (1)	Yes (1)	No (0)	No (0)
**Sucject 21**	Yes (1)	Yes (1)	Yes (1)	Yes (1)	Yes (1)	Yes (1)

**Sucject 22**	Yes (1)	No (0)	Yes (1)	Yes (1)	Yes (1)	No (0)
**Sucject 23**	Yes (1)	Yes (1)	No (0)	No (0)	Yes (1)	No (0)
**Sucject 24**	No (0)	Yes (1)	Yes (1)	Yes (1)	Yes (1)	Yes (1)
**Sucject 25**	No (0)	Yes (1)	Yes (1)	Yes (1)	Yes (1)	No (0)
**Sucject 26**	No (0)	Yes (1)	Yes (1)	Yes (1)	No (0)	Yes (1)
**Sucject 27**	No (0)	Yes (1)	Yes (1)	Yes (1)	Yes (1)	Yes (1)
**Sucject 28**	Yes (1)	No (0)	Yes (1)	Yes (1)	Yes (1)	Yes (1)
**Sucject 29**	Yes (1)	Yes (1)	Yes (1)	Yes (1)	Yes (1)	Yes (1)
**Sucject 30**	No (0)	Yes (1)	Yes (1)	Yes (1)	Yes (1)	No (0)
**Sucject 31**	Yes (1)	Yes (1)	Yes (1)	Yes (1)	Yes (1)	No (0)
**Sucject 32**	No (0)	Yes (1)	No (0)	No (0)	Yes (1)	No (0)
**Sucject 33**	Yes (1)	Yes (1)	Yes (1)	Yes (1)	Yes (1)	No (0)

**Sucject 34**	Yes (1)	Yes (1)	Yes (1)	Yes (1)	Yes (1)	Yes (1)
**Sucject 35**	Yes (1)	Yes (1)	Yes (1)	Yes (1)	Yes (1)	Yes (1)
**Sucject 36**	Yes (1)	No (0)	No (0)	No (0)	No (0)	Yes (1)
**Sucject 37**	Yes (1)	Yes (1)	Yes (1)	Yes (1)	Yes (1)	Yes (1)
**Sucject 38**	No (0)	No (0)	No (0)	Yes (1)	No (0)	Yes (1)
